# Associations of Green Tea and Rock Tea Consumption with Risk of Impaired Fasting Glucose and Impaired Glucose Tolerance in Chinese Men and Women

**DOI:** 10.1371/journal.pone.0079214

**Published:** 2013-11-18

**Authors:** Huibin Huang, Qiuxuan Guo, Changsheng Qiu, Baoying Huang, Xianguo Fu, Jin Yao, Jixing Liang, Liantao Li, Ling Chen, Kaka Tang, Lixiang Lin, Jieli Lu, Yufang Bi, Guang Ning, Junping Wen, Caijing Lin, Gang Chen

**Affiliations:** 1 Department of Endocrinology, Fujian Provincial Hospital Key Laboratory of Endocrinology, Fujian Medical University, Fuzhou, Fujian, China; 2 Department of Osteology, Wuyishan Municipal Hospital, Fujian Provincial Hospital, Wuyishan, Fujian, China; 3 Department of Endocrinology, Ningde Municipal Hospital, Fujian Medical University, Ningde, Fujian, China; 4 Department of Endocrinology, Ruijin Hospital, Shanghai Jiaotong University School of Medicine, Shanghai, China; 5 Department of Emergency, Fujian Provincial Hospital, Fujian Medical University, Fuzhou, Fujian, China; Pennington Biomedical Research Center, United States of America

## Abstract

**Objective:**

To explore the associations of green tea and rock tea consumption with risk of impaired fasting glucose (IFG) and impaired glucose tolerance (IGT).

**Methods:**

A multistage, stratified, cluster, random-sampling method was used to select a representative sample from Fujian Province in China. In total, 4808 subjects without cardiovascular disease, hypertension, cancer, or pancreatic, liver, kidney, or gastrointestinal diseases were enrolled in the study. A standard questionnaire was used to gather data on tea (green, rock, and black) consumption and other relevant factors. The assessment of impaired glucose regulation (IGR) was using 75-g oral glucose tolerance test (OGTT), the diagnostic criteria of normal glucose tolerance was according to American Diabetes Association.

**Results:**

Green tea consumption was associated with a lower risk of IFG, while rock tea consumption was associated with a lower risk of IGT. The adjusted odds ratios for IFG for green tea consumption of <1, 1–15, 16–30, and >30 cups per week were 1.0 (reference), 0.42 (95% confidence intervals (CI) 0.27–0.65), 0.23 (95% CI, 0.12–0.46), and 0.41 (95% CI, 0.17–0.93), respectively. The adjusted odds ratios for IGT for rock tea consumption of <1, 1–15, 16–30, and >30 cups per week were 1.0 (reference), 0.69 (95% CI, 0.48–0.98), 0.59 (95% CI, 0.39–0.90), and 0.64 (95% CI, 0.43–0.97), respectively. A U-shaped association was observed, subjects who consumed 16–30 cups of green or rock tea per week having the lowest odds ratios for IFG or IGT.

**Conclusions:**

Consumption of green or rock tea may protect against the development of type 2 diabetes mellitus in Chinese men and women, particularly in those who drink 16–30 cups per week.

## Introduction

Tea is consumed as a beverage worldwide, especially in Asia. Because of the high prevalence of tea consumption, even small effects of tea at a population level may have a large impact on public health. Tea, originates from the leaves of the plant *Camellia sinensis*, can be classified into three major types according to the level of fermentation: green tea (not fermented), oolong tea (partially fermented), and black tea (fully fermented) [1]. Rock tea is a kind of oolong tea produced in the Mount Wuyi region, Fujian, China, and is famous for its fragrance [2].

In recent years, tea has been studied for its potential to influence insulin activity, plasma glucose concentration, and the incidence of diabetes. However, evidence on the potential of tea to influence the development of diabetes is controversial. A meta-analysis reported that people who drank four or more cups of tea per day had a lower risk of type 2 diabetes [3]. A retrospective cohort study of 17413 Japanese adults also demonstrated that green tea consumption was associated with a lower risk of diabetes, whereas consumption of oolong or black tea was not [4]. Another prospective study conducted in ten European countries observed a linear inverse association between tea consumption and incidence of type 2 diabetes [5]. In contrast, other studies found no relationship between tea consumption and diabetes risk [6], [7].

Of these research studies, only a few examined the association between tea consumption and impaired glucose regulation, the so-called “prediabetic state”, which is identified as having impaired fasting glucose (IFG) and/or impaired glucose tolerance (IGT) [8]. Studies have shown that people with impaired glucose regulation have a significantly increased risk for diabetes mellitus [9], [10]. However, little is known on the effect of tea consumption on impaired glucose regulation. We therefore investigated the association of tea consumption (green and rock tea) with risk of impaired glucose regulation, using data from a cross-sectional study on Chinese Han people in Fujian province.

## Methods

### Study population

This was a cross-sectional study which was conducted between 2011–2012. Subjects registered were permanent residents of Fujian province. A multi-stage, stratified, cluster random-sampling method was used to select a representative sample. A total of 9995 people were registered in the study. Subjects were excluded if they had any of the following conditions: cardiovascular disease (i.e., myocardial infarction, heart failure, or stroke), hypertension, cancer, and pancreatic, liver, kidney, or gastrointestinal diseases, and whose data were not completed, since the presence of these diseases could have affected their diet, lifestyle and the absorption of tea. We also excluded participants who were diabetes or who drank black tea or other types of tea, because of the small sample size. In the end, 4808 subjects were enrolled. All the investigators received professional training before the investigations. The study received approval from the Endocrinology Branch of the Chinese Medical Association and all participants gave written informed consent.

### Data collection

The participants were asked to complete a standard questionnaire including age, gender, family history of diabetes, as well as medical histories, such as cardiovascular disease, hypertension, diabetes, cancer, and pancreatic, liver, kidney, and gastrointestinal diseases. Lifestyle factors were also recorded, for instance, diet, smoking habits, daily alcohol intake, physical activity, and sleep status. Dietary factors were assessed primarily by asking the participants whether and how they had consumed tea and other beverages such as milk, soybean milk, coffee, soda, and juice during the previous year. In particular, the participants were asked about tea consumption including the type of tea they drank (green, rock, or black) and how many cups of tea they consumed each day and how many days they consumed tea each week. The volume of one cup of tea was estimated to be equal to 150 mL. The cups consumed per day were multiplied by the number of days that tea was consumed each week to determine how many cups of tea were consumed each week. Four categories for frequency of tea consumption were created: <1 cup/week, 1–15 cups/week, 16–30 cups/week, and >30 cups/week. Milk, soybean milk, coffee, soda and juice drinkers were defined as those who drank once or more each week. Smoking status was assessed based on whether the participant was a past or present smoker, the number of cigarettes smoked daily, and the number of years that cigarettes had been smoked. Current smokers were those who smoked at least one cigarette per day. Former smokers were those who had smoked previously, but had not smoked for one year or more. Alcohol intake was assessed by asking the participants about their past and present drinking habits, including the quantity of drinks consumed each day and the number of years that they had been drinking alcohol. The participants were classified as never, former or current drinkers. Current drinkers were defined as those who had consumed alcohol once or more each week over a period of one year or longer. Former drinkers were distinguished from lifelong non-drinkers. Physical activity was measured by asking the participants whether they practiced leisure-time physical activity for at least 20–30 min twice or more each week. Sleep status was assessed based on whether the participants had insomnia or were using sleeping pills. Body weight and height were measured without shoes and in light clothing, and body mass index (BMI) calculated as weight (kg)/height (m)^2^. Waist circumference was measured at the middle point between the costal margin and iliac crest, and hip circumference was measured at the level of the trochanters. Waist to hip ratio (WHR) was calculated as the ratio of waist to hip circumference. Blood pressure was measured twice in the right arm, in the sitting position, using a manual sphygmomanometer, after the subjects had been resting for 30 min. The mean of the two readings was used in the analyses.

### Laboratory measurements

5 ml blood with and without anticoagulant (sodium fluoride + potassium oxalate, 1∶3) were taken from subjects who had fasted for at least 10 h. All subjects were administered 75-g oral glucose tolerance test (OGTT). After 30 and 120 min, 5 ml blood with anticoagulant was drawn from subjects. Levels of blood glucose, total cholesterol (TC), triglycerides (TG) and high-density lipoprotein cholesterol (HDL-C) were tested using the glucose oxidase method, colorimetric enzyme assays, glycerol phosphate enzymatic oxidation assay, and end point colorimetry, respectively. Low-density lipoprotein cholesterol (LDL-C) was calculated by the formula of Friedewald et al [11].

### Diagnostic categories

According to the diagnostic criteria of the American Diabetes Association [8], normal glucose tolerance was defined as fasting plasma glucose (FPG) <5.6 mmol/L and 2-h post load plasma glucose (2hPG) <7.8 mmol/L with no previous diagnosis of diabetes or impaired glucose regulation. Impaired fasting glucose (IFG) was defined as 5.6≤ FPG <7.0 mmol/L. Impaired glucose tolerance (IGT) was defined as 7.8≤2 h PG <11.1 mmol/L. Diagnosis of diabetes was based on FPG ≥7.0 mmol/L and/or 2h PG ≥11.1 mmol/L. Hypertension was defined as systolic blood pressure ≥140 mmHg and/or diastolic blood pressure ≥90 mmHg or having been diagnosed with hypertension or taking anti-hypertension treatment. Dyslipidemia was defined as self-reported current treatment with cholesterol-lowering medication or having one or more of the following: TC ≥5.17 mmol/L, TG ≥1.69 mmol/L, HDL-C ≤1.03 mmol/L, or LDL-C ≥3.38 mmol/L [12]. Four categories for the frequency of green or rock tea consumption were created: <1 cup/week, 1–15 cups/week, 16–30 cups/week, and >30 cups/week. Based on the smoking status, subjects were classified as never, former, and current smokers, and based on the alcohol intake, they were classified as never, former, and current drinkers. Subjects were categorized as inactive and active based on the level of physical activity; and based on the sleep status, subjects were classified as those who slept well and those who did not. BMI was classified as normal, overweight (25.0–29.9 kg/m^2^), and obesity (≥30.0 kg/m^2^) according to the WHO criteria [13].

### Statistic analysis

EpiData software (EpiData Association, Odense, Denmark) was used to establish the database and the statistical program SPSS 19.0 (SPSS Inc., Chicago, IL, USA) used for the statistical analyses. Continuous variables were expressed as mean (standard deviation) for normally distributed variables and as median (interquartile ranges) for variables with a non-normal distribution. Categorical variables were expressed as a percentage. Differences in means, medians and percentage of baseline variables across the green tea and rock tea consumption categories were statistically tested by one-way ANOVA, the Kruskal–Wallis test and the chi-square test respectively. Univariate analyses of general linear models were used to compare differences in the mean concentrations of fasting and 2-h post load plasma glucose according to the consumption of each tea in subjects with normal glucose tolerance. As green and rock tea consumption did not overlap, we did not mutually adjust for their consumption. The odds ratios (OR) and 95% confidence intervals of IFG and IGT in relation to levels of green and rock tea consumption were obtained by multiple logistic regression analysis adjusted for confounding variables, using normal glucose tolerance as the reference group. An OR value <1 was regarded as a protective factor. All *p*-values <0.05 from two-sided hypotheses were considered as statistically significant.

## Results

Of the 4808 subjects, 1242 (25.83%) had IFG and 648 (13.48%) had IGT. The baseline characteristics of the subjects grouped according to the level of green and rock tea consumption are shown in Tables 1 and 2, respectively. Subjects who drank green tea were older than those who did not, while subjects who drank rock tea were younger than those who did not. Subjects who consumed both teas were more likely to be men, with the percentage of men increasing with higher tea consumption. The percentage of former smokers, current smokers, and subjects who drank alcohol was significantly higher for both tea consumptions than that of subjects who did not consume tea. Subjects who drank rock tea also had a higher percentage for a family history of diabetes, were more likely to drink milk and soybean milk, and less likely to participate in physical activity. Although consumption of the two teas did not cause significant improvements in lipid metabolism, BMI, or waist-hip ratio, the effect of an unhealthy lifestyle should not be excluded. The percentage of subjects who consumed coffee, soda, or juice was very low, and because there was no significant difference between the groups, we did not adjust for these variables in the later analyses.

**Table 1:** Baseline characteristics grouped according to weekly consumption of green tea.

Variable	Green tea consumption (cups per week)				*p* value
	<1	1–15	16–30	>30	
	(n = 2934)	(n = 215)	(n = 140)	(n = 68)	
Age (year)	53 (46–61)	56 (47–66)	60 (50–71)	59 (48–66)	<0.001
Sex (male/female) %	30.1/69.9	49.8/50.2	66.9/33.1	70.7/29.3	<0.001
HDL –C (mmol/L)	1.35 (1.13–1.60)	1.25 (1.07–1.53)	1.24 (1.08–1.47)	1.28 (1.05–1.54)	<0.001
LDL –C (mmol/L)	2.89 (0.86)	2.88 (0.86)	3.00 (0.82)	2.94 (0.81)	0.54
TC (mmol/L)	4.95 (1.11)	4.96 (1.19)	5.08 (0.99)	5.08 (1.03)	0.49
TG (mmol/L)	1.15 (0.85–1.64)	1.22 (0.88–1.62)	1.25 (0.94–1.84)	1.21 (0.82–1.89)	0.13
Family history of diabetes (%)	9.9	12.1	13.1	8.6	0.48
Milk drinkers (%)	41.8	45.1	43.8	34.5	0.48
Soybean milk drinkers (%)	76.6	79.1	83.8	82.8	0.14
Coffee drinkers (%)	1.6	2.8	1.5	1.7	0.63
Soda drinkers (%)	2.7	5.1	4.6	5.2	0.11
Juice drinkers (%)	5.4	4.7	3.8	5.2	0.85
Current smokers (%)	13.7	23.7	36.9	41.4	<0.001
Former smokers (%)	4.6	10.7	14.6	17.2	<0.001
Current drinkers (%)	10.2	18.6	22.3	31.0	<0.001
Sleep well (%)	81.7	81.9	80.8	79.3	0.96
Physically inactive (%)	15.5	6.0	4.6	5.2	<0.001
BMI (kg/m^ 2^)	23.52 (3.19)	23.82 (3.21)	24.07 (3.55)	23.48 (3.55)	0.19
WHR	0.86 (0.07)	0.87 (0.06)	0.89 (0.06)	0.90 (0.13)	<0.001

Data are expressed as mean (SD), median (interquartile ranges), or percentages. HDL-C, high-density lipoprotein-cholesterol; LDL-C, low-density lipoprotein-cholesterol; TC, total cholesterol; TG, triglycerides; BMI, body mass index; WHR, waist to hip ratio.

**Table 2:** Baseline characteristics grouped according to weekly consumption of rock tea.

Variable	Rock tea consumption (cups per week)				*p* value
	<1	1–15	16–30	>30	
	(n = 2934)	(n = 553)	(n = 416)	(n = 482)	
Age (year)	53 (46–61)	48 (44–55)	48 (43–55)	48 (44–55)	<0.001
Sex (male/female) %	30.1/69.9	58.8/41.2	67.8/32.2	79.5/20.5	<0.001
HDL –C (mmol/L)	1.35 (1.13–1.60)	1.38 (1.19–1.57)	1.36 (1.16–1.58)	1.32 (1.14–1.55)	0.41
LDL –C (mmol/L)	2.89 (0.86)	2.93 (0.77)	3.03 (0.81)	3.01 (0.75)	0.001
TC (mmol/L)	4.95 (1.11)	5.04 (0.93)	5.22 (1.06)	5.14 (0.87)	<0.001
TG (mmol/L)	1.15 (0.85–1.64)	1.36 (0.94–2.03)	1.44 (1.03–2.06)	1.47 (1.04–2.23)	<0.001
Family history of diabetes (%)	9.9	17.4	20.4	13.9	<0.001
Milk drinkers (%)	41.8	51.2	52.4	52.7	<0.001
Soybean milk drinkers (%)	76.6	76.1	81.3	81.3	0.02
Coffee drinkers (%)	1.6	2.9	2.4	1.9	0.17
Soda drinkers (%)	2.7	3.8	2.9	2.9	0.59
Juice drinkers (%)	5.4	6.3	5.8	8.1	0.12
Current smokers (%)	13.7	37.3	44.5	55.8	<0.001
Former smokers (%)	4.6	9.4	8.4	7.3	<0.001
Current drinkers (%)	10.2	30.2	28.4	38.4	<0.001
Sleep well (%)	81.7	87.2	87.3	88.2	<0.001
Physically inactive (%)	15.5	23.0	18.5	28.2	<0.001
BMI (kg/m^ 2^)	23.52 (3.19)	23.91 (2.75)	24.34 (3.00)	24.54 (3.13)	<0.001
WHR	0.86 (0.07)	0.87 (0.09)	0.88 (0.06)	0.88 (0.06)	<0.001

Data are expressed as mean (SD), median (interquartile ranges), or percentages. HDL-C, high-density lipoprotein-cholesterol; LDL-C, low-density lipoprotein-cholesterol; TC, total cholesterol; TG, triglycerides; BMI, body mass index; WHR, waist to hip ratio.

Next we used univariate analyses of general linear models to compare differences in the mean concentration of FPG and 2hPG glucose according to the consumption of each tea in subjects with normal glucose tolerance (Table 3). Model 1 (adjusted for age and gender) and model 2 (adjusted for age, gender, serum lipids, family history of diabetes, consumption of milk, consumption of soybean milk, smoking status, alcohol intake, sleep status, physical activity, BMI, and waist-hip ratio), both showed that subjects who consumed green tea on a weekly basis had lower FPG levels, while subjects who consumed rock tea on a weekly basis had lower 2hPG levels. The reductions in FPG and 2hPG were more pronounced in subjects who consumed 16–30 cups of green or rock tea per week.

**Table 3:** Plasma glucose concentrations grouped according to weekly consumption of green tea or rock tea.

	Tea consumption (cups per week)				*p* value
	<1	1–15	16–30	>30	
	Model 1				
Green tea					
Fasting PG (mmol/L)	5.03 (0.01)	4.74 (0.04)*	4.61 (0.05)*	4.70 (0.07)*	<0.001
2-h post load PG (mmol/L)	5.88 (0.02)	5.97 (0.09)	6.04 (0.12)	5.94 (0.18)	0.52
Rock tea					
Fasting PG (mmol/L)	5.03 (0.11)	5.08 (0.02)	5.09 (0.02)	5.05 (0.02)	0.137
2-h post load PG (mmol/L)	5.81 (0.02)	5.69 (0.06)	5.65 (0.06)*	5.68 (0.06)	0.048
	Model 2				
Green tea					
Fasting PG (mmol/L)	5.02 (0.01)	4.76 (0.03)*	4.63 (0.05)*	4.73 (0.07)*	<0.001
2-h post load PG (mmol/L)	5.89 (0.02)	5.93 (0.09)	6.00 (0.12)	5.93 (0.18)	0.812
Rock tea					
Fasting PG (mmol/L)	5.05 (0.01)	5.07 (0.02)	5.08 (0.02)	5.02 (0.03)	0.306
2-h post load PG (mmol/L)	5.82 (0.02)	5.70 (0.06)	5.63 (0.06)*	5.65 (0.06)*	0.023

Subjects with normal glucose tolerance were included in the analysis. Data are expressed as mean (SD). Model 1 was adjusted for age and gender. Model 2 was adjusted for age, gender, HDL-C, LDL-C, TC, TG, family history of diabetes, consumption of milk, consumption of soybean milk, smoking, consumption of alcohol, sleep, physical activity, BMI, and waist to hip ratio. *Compared with drinking <1 cup per week, *p*<0.05.

Tables 4 and 5 show the multivariable-adjusted ORs and 95% confidence interval (CI) for IFG and IGT according to the level of green or rock tea consumption, with normal glucose tolerance acting as the reference group. Two different models were constructed respectively. The first model was adjusted for age, gender, and level of tea consumption. The second model included the variables in model 1, in addition to being adjusted for dyslipidemia, family history of diabetes, consumption of milk, consumption of soybean milk, smoking status, alcohol intake, physical activity, sleep status, BMI, and waist-hip ratio. Compared with subjects who never consumed tea, subjects who consumed green tea had a reduced risk of IFG, with the adjusted ORs for IFG in the green tea consumption categories of <1, 1–15, 16–30, and >30 cups per week being 1.0 (reference), 0.42 (95% CI, 0.27–0.65), 0.23 (95% CI, 0.12–0.46), and 0.41 (95% CI, 0.17–0.93), respectively (see in Tabel4). Subjects who consumed rock tea had a reduced risk for IGT, with the adjusted ORs of IGT in the rock tea consumption categories of <1, 1–15, 16–30, and >30 cups per week being 1.0 (reference), 0.69 (95% CI, 0.48–0.98), 0.59 (95% CI, 0.39–0.90), and 0.64 (95% CI, 0.43–0.97) (seen in Tabel5). A U-shaped association was observed respectively, subjects who consumed 16–30 cups of green or rock tea per week having the lowest ORs for IFG or IGT.

**Table 4:** Odds ratio for glucose tolerance status grouped according to the level of green tea consumption.

Glucose tolerance status		Green tea consumption (cups per week)			
		<1	1–15	16–30	>30
Normal	N	1744	140	87	37
IFG	N	752	25	20	17
	Model1(OR,95% CI)	1.00(reference)	0.38 (0.24–0.58)	0.22 (0.11–0.43)	0.38 (0.16–0.86)
	* p* value		<0.001	<0.001	0.02
	Model2(OR,95% CI)	1.00(reference)	0.42 (0.27–0.65)	0.23 (0.12–0.46)	0.41 (0.17–0.93)
	* p* value		<0.001	<0.001	0.034
IGT	N	438	50	33	14
	Model1(OR,95% CI)	1.00(reference)	1.26 (0.88–1.79)	1.21 (0.78–1.88)	1.46 (0.76–2.79)
	* p* value		0.193	0.385	0.247
	Model2(OR,95% CI)	1.00(reference)	1.11 (0.77–1.59)	1.08 (0.69–1.68)	1.19 (0.61–2.30)
	* p* value		0.564	0.731	0.606

Abbreviations: CI, confidence interval; IFG, impaired fasting glucose; IGT, impaired glucose tolerance.

Model1: Adjusted for age, gender, and level of green tea consumption.

Model2: Adjusted for age, gender, level of green tea consumption, dyslipidemia, family history of diabetes, consumption of milk, consumption of soybean milk, smoking status, consumption of alcohol, physical activity, sleep status, BMI, and waist to hip ratio.

**Table 5:** Odds ratio for glucose tolerance status grouped according to the level of rock tea consumption.

Glucose tolerance status		Rock tea consumption (cups per week)			
		<1	1–15	16–30	>30
Normal	N	1744	345	267	298
IFG	N	752	162	118	148
	Model1(OR,95% CI)	1.00(reference)	1.05 (0.84–1.30)	0.95 (0.75–1.22)	1.04 (0.82–1.31)
	* p* value		0.656	0.723	0.724
	Model2(OR,95% CI)	1.00(reference)	0.99 (0.79–1.24)	0.90 (0.70–1.16)	0.91 (0.71–1.16)
	* p* value		0.967	0.448	0.418
IGT	N	438	46	31	36
	Model1(OR,95% CI)	1.00(reference)	0.71 (0.51–0.99)	0.65 (0.43–0.97)	0.70 (0.47–1.03)
	* p* value		0.044	0.036	0.071
	Model2(OR,95% CI)	1.00(reference)	0.69 (0.48–0.98)	0.59 (0.39–0.90)	0.64 (0.43–0.97)
	* p* value		0.039	0.015	0.034

Abbreviations: CI, confidence interval; IFG, impaired fasting glucose; IGT, impaired glucose tolerance.

Model1: Adjusted for age, gender, and level of rock tea consumption.

Model2: Adjusted for age, gender, level of rock tea consumption, dyslipidemia, family history of diabetes, consumption of milk, consumption of soybean milk, smoking status, consumption of alcohol, physical activity, sleep status, BMI, and waist to hip ratio.

## Discussion

This study demonstrated the inverse associations between green tea consumption and IFG, and between rock tea consumption and IGT. These inverse associations appeared to be more evident when subjects consumed 16–30 cups of tea per week. People with IFG and IGT have a higher risk of developing diabetes mellitus. Our study adds to increasing evidence that tea consumption may provide protection against the development of type 2 diabetes. However, to date, the relationship between tea consumption and diabetes has been inconsistent. Recent epidemiologic studies have suggested that tea consumption reduces the risk of type 2 diabetes [5], [14], [15], whereas other studies have shown no effect [6], [7] Studies have also been conducted on different types of tea (green, oolong, and black), although these studies have also produced conflicting results. A retrospective cohort study of 17413 Japanese adults showed that green tea consumption was associated with a lower diabetes risk, whereas oolong or black tea consumption was not [4]. However, Yamaji et al. reported no clear association between green tea consumption and diabetes [16]. A clinical trial reported that consumption of oolong tea decreased the plasma glucose level in patients with type 2 diabetes [17]. Whereas another study reported long-term consumption of oolong tea may be a predictive factor for new onset diabetes [18]. The Singapore Chinese Health Study also demonstrated that regular consumption of black tea, but not of green tea, was associated with a lower risk of type 2 diabetes [19]. For assessment of type 2 diabetes, the majority of these studies used self-reported questionnaires and/or registers of diabetic patients receiving treatment, with only a few studies adopted a standard OGTT.

Although tea contains numerous compounds, its main biological effects are attributable to polyphenols, especially flavonoids [20]. Catechins, theaflavins, and thearubigins are the most prominent flavonoids in tea [5], [20]. Tea also contains caffeine, which is also one of the important components of it [17]. According to the level of fermentation, it can be classified into green tea (not fermented), oolong tea (partially fermented) and black tea (fully fermented) [1]. The components of these three types of teas are different and have variable bioactivities. Green tea comes from steamed fresh leaves, which contain a class of flavonoids known as catechins, comprised of epigallocatechin gallate (EGCG), epicatechin gallate, and gallocatechin gallate. EGCG is thought to be the most pharmacologically active of these catechins [21], [22]. Rock tea is a kind of oolong tea, which is semi-fermented. During the production of rock tea, the majority of catechins are transformed to theaflavins and thearubigins [2], [22]. A recent comparative study of different teas reveals important findings: the levels of EGCG and total catechins are in the order of green tea > oolong tea > black tea; the level of caffeine in different teas is in the order of black tea > oolong tea > green tea; the induction of apoptosis is in the order of green tea > oolong tea > black tea [22].

Our results show that consumption of green tea significantly decreased FPG concentrations, and consumption of rock tea significantly decreased 2hPG concentrations, and the same results were observed in subjects with normal glucose tolerance after adjustment for confounding variables. These results may be explained by the level of tea fermentation and the different mechanism of IFG and IGT. The chemical composition and bioactivity of the teas is known to change with the level of fermentation, IFG and IGT are also two different states in insulin resistance and insulin secretion [23]. IFG have severe hepatic insulin resistance with normal or near-normal muscle insulin resistance, while IGT have marker muscle insulin resistance with only mild hepatic insulin resistance. Both of them are characterized by a reduction in early-phase insulin secretion, while subject with IGT also have impaired late-phase insulin secretion. Both conditions contribute to disturbances in glucose homeostasis and the development of type 2 diabetes [24]. In our study, green tea particularly lowered the risk of IFG, probably as a result of its high catechin content, especially EGCG. EGCG has insulin mimetic effect, it decreases hepatic glucose production, and increases tyrosine phosphorylation of the insulin receptor and insulin receptor substrate-1 (IRS-1). In addition, it controls gluconeogenesis by inhibiting the expression of genes such as phosphoenolpyruvate carboxykinase *(PEPCK*) and glucose-6-phosphatase *(G6Pase*), and it can also ameliorate cytokine-induced β-cell damage and improve insulin sensitivity [3], [25]–[27]. Studies in rats have shown green tea extracts also reduce fasting hyperglycemia [28], regulate the expression of genes involved in glucose uptake and the insulin signal transduction pathways [29], scavenge free radicals, reduce oxidative stress, and have an anti-diabetic effect [30]. Compared with green tea, rock tea is semi-fermented, and during fermentation, the majority of catechins are transformed to theaflavins and thearubigins [2], [22]. In our study, rock tea particularly lowered the risk of IGT, it could be speculated that it may inhibit postprandial hyperglycemia and protect against the development of type 2 diabetes. Matsui et al. reported that theaflavins have an anti-hyperglycemic effect, and it may delay or inhibit glucose production at the intestine through the inhibition of α-glucosidase activity [31]. Williamson et al. also showed that extracts from tea, especially theaflavins, but not EGCG, were effectively inhibit human salivary α-amylase by a non-competitive mechanism [32]. The comparative study of different teas from Taiwan mentioned above also showed that rock tea (a kind of oolong tea) contains more caffeine than green tea. It has been reported that caffeine is involved in the regulation of glucose metabolism in the skeletal muscle [33], increases glucose transporter IV expression [34], and has anti-diabetic activity [35]. Epidemically studies also revealed the inverse association between caffeine intake and risk of diabetes [4], [16]. However, the specific mechanism of the antihyperglycemic effect of rock tea remains unclear and requires further study.

In present study, we also observed that the inverse associations between green tea consumption and IFG, and rock tea consumption and IGT were more pronounced when subjects drank 16–30 cups of tea per week. However, the level of tea consumption required to reduce the risk of type 2 diabetes remains controversial. A meta-analysis reported that subjects who drank four or more cups of tea per day had a 20% lower risk of developing diabetes than those who drank less or none [3]. Another prospective study reported a linear inverse association between tea consumption and incidence of type 2 diabetes, although this protective effect was not restricted to subjects who drank >4 cups of tea per day, with lower doses also reducing the risk of diabetes [5]. Another cross-sectional study revealed that long-term tea consumption (1–2 cups per day) was associated with a lower risk of diabetes [15]. It is possible that differences in study design, the type of tea consumed, the cup size used, the sample size under investigation, and lifestyle may have contributed to these conflicting results. In addition, a review article suggested that although tea has numerous benefits, it is necessary to consider the adverse effects that may accompany heavy consumption of tea or catechins [36]. For example, it has been reported that high doses of EGCG act as prooxidants, which can lead to normal cell apoptosis [37], [38].

The strengths of the present study include the use of oral glucose tolerance tests to evaluate the relationship between tea consumption and impaired glucose regulation, and specific assessments of green and rock tea, whereas other studies have typically grouped the tea types into the one category. The study had several limitations. First, because of the cross-sectional design of the study, we were unable to determine cause- and-effect relationships. Second, tea consumption was assessed using self-reported questionnaires, and therefore, misclassifications may have occurred. However, these misclassifications would tend to underestimate the effect of tea consumption. Third, the year of tea consumption may influence the effect of tea on glucose regulation, but these factors were not controlled in our studies. Forth, we did not distinguish between drinking tea before or after meals, which may affect the bioactivities of the compounds in tea and change appetite and absorption of nutrients. However, almost all of the earlier studies that explored the relationship between tea and diabetes did not adjusted for this factor, and in China, the majority of people drink tea after meals. Fifth, we did not use a validated food frequency questionnaire to evaluate food and drink intake. Sixth, the study population included Chinese men and women living in Fujian, whose lifestyle is different from people living in Western countries. For example, the consumption of coffee, soda, and juice is relatively low in China compared with that in Western countries. Therefore, whether or not our results can be generalized to other populations requires further study.

In conclusion, this study found inverse associations between green tea consumption and IFG, and between rock tea consumption and IGT, in Chinese women and men. These inverse associations were more pronounced in subjects who drank 16–30-cups of tea each week. These findings are important because of the prevalent consumption of tea and the growing epidemic of type 2 diabetes. Further human studies, especially clinical trials, are needed to investigate the role of green and rock tea consumption in relationship to the risk of glucose intolerance.
